# Protein kinase CK2 is widely expressed in follicular, Burkitt and diffuse large B-cell lymphomas and propels malignant B-cell growth

**DOI:** 10.18632/oncotarget.3446

**Published:** 2015-01-31

**Authors:** Marco Pizzi, Francesco Piazza, Claudio Agostinelli, Fabio Fuligni, Pietro Benvenuti, Elisa Mandato, Alessandro Casellato, Massimo Rugge, Gianpietro Semenzato, Stefano A. Pileri

**Affiliations:** ^1^ Department of Medicine, Surgical Pathology and Cytopathology Unit, DIMED University of Padua, Padua, Italy; ^2^ Department of Medicine, Hematology and Clinical Immunology Branch, DIMED University of Padua, Padua, Italy; ^3^ Venetian Institute of Molecular Medicine (VIMM), Padua, Italy; ^4^ Department of Experimental, Hematopathology and Hematology Sections, Diagnostic and Specialty Medicine, S. Orsola-Malpighi Hospital, University of Bologna, Bologna, Italy

**Keywords:** CK2, Non-Hodgkin Lymphoma, CX-4945, B-cell

## Abstract

Serine-threonine kinase CK2 is highly expressed and pivotal for survival and proliferation in multiple myeloma, chronic lymphocytic leukemia and mantle cell lymphoma. Here, we investigated the expression of α catalytic and β regulatory CK2 subunits by immunohistochemistry in 57 follicular (FL), 18 Burkitt (BL), 52 diffuse large B-cell (DLBCL) non-Hodgkin lymphomas (NHL) and in normal reactive follicles. *In silico* evaluation of available Gene Expression Profile (GEP) data sets from patients and Western blot (WB) analysis in NHL cell-lines were also performed. Moreover, the novel, clinical-grade, ATP-competitive CK2-inhibitor CX-4945 (Silmitasertib) was assayed on lymphoma cells. CK2 was detected in 98.4% of cases with a trend towards a stronger CK2α immunostain in BL compared to FL and DLBCL. No significant differences were observed between Germinal Center B (GCB) and non-GCB DLBCL types. GEP data and WB confirmed elevated CK2 mRNA and protein levels as well as active phosphorylation of specific targets in NHL cells. CX-4945 caused a dose-dependent growth-arresting effect on GCB, non-GCB DLBCL and BL cell-lines and it efficiently shut off phosphorylation of NF-κB RelA and CDC37 on CK2 target sites. Thus, CK2 is highly expressed and could represent a suitable therapeutic target in BL, FL and DLBCL NHL.

## INTRODUCTION

Protein kinase CK2 is a tetrameric enzyme, composed by two catalytic (α and/or α') and two regulatory (β) subunits. CK2 and its orthologs are highly conserved throughout evolution and are involved in the phosphorylation of hundreds of protein targets (20% of human phosphoproteome) [1].

The wide spectrum of possible phosphorylation substrates justifies the pleiotropic roles of CK2 in cell biology. *In vitro* studies have demonstrated that CK2 is involved in cell cycle regulation, gene expression, protein translation, DNA repair and programmed cell-death [2]. CK2 is also known to be a master regulator of embryonic development, as it is involved in the mid-gestational morphogenesis of heart, brain, pharyngeal arch and somites [3, 4].

The central role of CK2 in several physiological processes is paralleled by its deregulation in many (solid and hematological) tumors [5, 6]. The over-expression of such kinase has indeed been documented in prostate, breast, lung, head and neck and colon carcinomas [7-13]. In these tumors, CK2 up-regulation has been associated with increased tumor-cell survival and worse overall prognosis. Of note, treatment of cancer cells with CX-4945 (Silmitasertib, a novel recently developed clinically grade ATP-competitive highly selective CK2-inhibitor) can induce a significant reduction of cell-growth and survival [11, 14, 15]. A common viewpoint on how CK2 sustains cancer cell growth relies on the “non oncogene addiction” process, whereby cancer cells exploit the advantages to keep up-regulated a critical “transversal” protein able to propel different oncogenic pathways [6].

Among lymphoproliferative disorders, CK2 over-expression has been reported in both precursor lymphoid (T-and B-Acute Lymphoblastic Leukemia) and mature B-cell neoplasms [5]. The latter encompass B-Chronic Lymphocytic Leukemia (B-CLL) [16, 17], Mantle Cell Lymphoma (MCL) [18] and Plasma Cell Myeloma (PCM) [18, 19]. As for carcinoma cell-lines, *in vitro* and *in vivo* pre-clinical studies from our and other groups have indicated that first generation CK2 inhibitors as well as the newer CX-4945 have the potential to be novel therapeutic tools for the treatment of high CK2-expressing B cell tumors [16, 17, 20].

To fill a gap about to what extent is CK2 expressed in NHL and as to whether its inhibition could affect lymphoma cell viability, the present study evaluated CK2 mRNA and protein levels in the commonest forms of B-cell NHL: Follicular Lymphoma (FL), Diffuse Large B-Cell Lymphoma (DLBCL) and Burkitt Lymphoma (BL). CK2 protein expression was investigated by immunohistochemistry in a series of 127 formalin-fixed paraffin-embedded (FFPE) biopsy samples. The obtained data were subsequently confirmed by checking CK2 mRNA levels from a repository of published cDNA microarray data available in the Oncomine database [21]. Immunohistochemical and molecular results were further validated by WB analysis on matched NHL cell-lines. To explore the effects and a possible therapeutic role for CK2 inactivation in the treatment of such lymphoid malignancies, CX-4945 was used in *in vitro* cell viability assays and WB analysis of the phosphorylation of CK2 target site on NF-κB RelA and CDC37, demonstrating that CX-4945 is highly effective in inducing cell growth arrest of GCB and non-GCB type DLBCL as well as BL cell lines and in inhibiting CK2 kinase activity directed towards pivotal signaling molecules.

## RESULTS

### CK2α and CK2β protein expression in primary NHL tissues

**Figure 1:**
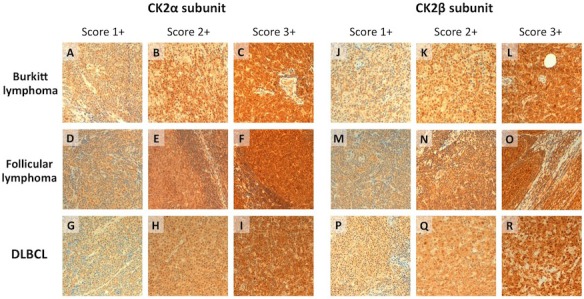
CK2α and CK2β expression by immunohistochemistry in FL, BL and DLBCL Representative immunohistochemical features of the considered lymphoproliferative lesions. Both BL (A-C; J-L), FL (D-F; M-O), and DLBCL (G-I; P-R) displayed consistent positivity for the regulatory and catalytic CK2 subunits. In all the considered lymphoma subtypes, variable immunohistochemical positivity (from score 1+ to score +3) was observed (H&E and immunoperoxidase stain; original magnification, x20).

Immunohistochemical analysis confirmed that in normal lymphoid tissue (tonsil) a moderate expression of CK2α and CK2β is confined to the follicular area whereas only faint reactivity could be detected in the mantle zone (Figure S1 and 18]). Overall, 98.4% (125/127) of the NHL cases analyzed disclosed some degree of CK2 expression (Figure 1, Table 1). Most of the lymphoma samples displayed high nuclear/cytoplasmic protein expression. In particular, a moderate to strong positivity (high-expression group: score ≥2+) was documented in more than 88% of cases (CK2α and CK2β score ≥2+: 88.2% (112/127) for both subunits) (details in Figure S2).

Compared to FL and DLBCL, BL was more frequently associated with moderate-to-strong CK2α expression (trend of association, Fisher's exact test). In particular, CK2α score ≥2+ was recorded in 94.4% (17/18) of BLs and in 86.0% (49/57) and 88.5% (46/52) of FLs and DLBCLs, respectively. Less marked differences were observed in CK2β expression patterns (Table 1).

As for DLBCL, CK2 subunits were moderately-to-strongly expressed in both GCB and non-GCB subtypes [CKα score ≥2+: 90.9% (20/22) of non-GCB DLBCL and 86.7% (26/30) of GCB-DLBCL; CK2β score ≥2+: 90.9% (20/22) of non-GCB DLBCL and 90.0% (27/30) of GCB-DLBCL; no statistically significant differences, Fisher's exact test; Table 1].

CK2 subunits were equally expressed in Bcl2-positive and Bcl2-negative FL. In particular, moderate-to-strong positivity for CK2α was recorded in 85.7% (24/28) of Bcl2-positive and in 86.2% (25/29) of Bcl2-negative FL (no statistically significant differences, Fisher's exact test). Similar results were obtained for the CK2β subunit (Table 1). No significant differences in CK2 expression were recorded among FL of different grades (Table 2).

**Table 1:** CK2α and CK2β expression in BL, FL and DLBCL

Lymphoma	Type (number)	CK2α				CK2β			
		0	1+	2+	3+	0	1+	2+	3+
Burkitt (BL)	Sporadic (18)	0% (0/18)	5.6% (1/18)	61.1% (11/18)	33.3% (6/18)	0% (0/18)	11.1% (2/18)	55.6% (10/18)	33.3% (6/18)
Follicular (FL)	Bcl2-positive (28)	0% (0/28)	14.3% (4/28)	46.4% (13/28)	39.3% (11/28)	0% (0/28)	14.3% (4/28)	42.9% (12/28)	42.9% (12/28)
	Bcl2-negative (29)	0% (0/29)	13.8% (4/29)	58.6% (17/29)	27.6% (8/29)	0% (0/29)	13.8% (4/29)	69.0% (20/29)	17.2% (5/29)
	Total cases (57)	0% (0/57)	14.0% (8/57)	52.6% (30/57)	33.3% (19/57)	0% (0/57)	14.0% (8/57)	56.1% (32/57)	29.8% (17/57)
DLBCL	GCB-type (30)	3.3% (1/30)	10.0% (3/30)	73.3% (22/30)	13.3% (4/30)	3.3% (1/30)	6.7% (2/30)	73.3% (22/30)	16.7% (5/30)
	non GCB-type (22)	4.5% (1/22)	4.5% (1/22)	59.1% (13/22)	31.8% (7/22)	4.5% (1/22)	4.5% (1/22)	72.7% (16/22)	18.2% (4/22)
	Total cases (52)	3.8% (2/52)	7.7% (4/52)	67.3% (35/52)	21.2% (11/52)	3.8% (2/52)	5.8% (3/52)	73.1% (38/52)	17.3% (9/52)

Abbreviations. Non-GCB= non Germinal Center B-cell ; GCB= Germinal Center B-cell; DLBCL= Diffuse Large B-Cell Lymphoma.

**Table 2:** CK2α and CK2β expression by FL grade

Follicular Lymphoma Grade	CK2α				CK2β			
	0	1+	2+	3+	0	1+	2+	3+
Grade 1 (13 cases)	0 (−)	2/13 (15.4%)	7/13 (53.8%)	4/13 (30.8%)	0 (−)	2/13 (15.4%)	7/13 (53.8%)	4/13 (30.8%)
Grade 2 (26 cases)	0 (−)	3/26 (11.5%)	13/26 (50.0%)	10/26 (38.5%)	0 (−)	3/26 (11.5%)	14/26 (53.8%)	9/26 (34.6%)
Grade 3 (18 cases)	0 (−)	3/18 (16.7%)	10/18 (55.6%)	5/18 (27.8%)	0 (−)	3/18 (16.7%)	11/18 (61.1%)	4/18 (22.2%)
Total	0 (−)	8/57 (14.0%)	30/57 (52.6%)	19/57 (33.3%)	0 (−)	8/57 (14.0%)	32/57 (56.1%)	17/57 (29.8%)

### GEP analysis of CK2 mRNA expression in NHL

**Figure 2:**
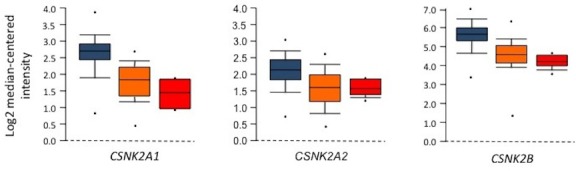
Gene expression data for CK2 subunits in FL, BL and DLBCL *CSNK2A1*, *CSNK2A2* and *CSNK2B* mRNA expression levels from Basso *et al.* data set are considered [22]. Box plots show consistent mRNA expression in BL (light blu), DLBCL (yellow) and FL (red), with significantly higher mRNA levels in BL compared to both DLBCL and FL (*CSNK2A1* expression data: BL *versus* DLBCL: t test, P = 7.32 E-12; BL *versus* FL: t test, P = 0.00038; DLBCL *versus* FL: t test, P = 0.07449; *CSNK2A2* expression data: BL *versus* DLBCL: t test, P = 1.41 E-7; BL *versus* FL: t test, P = 0.0002; DLBCL *versus* FL: t test, P = 0.78113; *CSNK2B* expression data: BL *versus* DLBCL: t test, P = 3.06 E-9; BL *versus* FL: t test, P = 3.71 E-6; DLBCL *versus* FL: t test, P = 0.03349).

To evaluate whether differences in CK2 protein expression were also present at the mRNA level, the mRNA signatures of both catalytic (*CSNK2A1* and *CSNK2A2*) and regulatory (*CSNK2B*) subunits were assessed, by checking available gene expression profiles (GEP) data sets in the Oncomine database [21]. GEP of the available study [22] confirmed the trend of the immunohistochemical results, with high *CSNK2A1*, *CSNK2A2* and *CSNK2B* mRNA levels in all the considered lymphoid tumors. In particular, BL featured higher *CSNK2A1* and *CSNK2A2* expression compared to both FL and DLBCL (Student's *t-*test; p<0.001). The same results were obtained for *CSNK2B* (Student's *t-*test; p<0.001) (Figure 2). Differences in the mRNA signatures of FL and DLBCL were not statistically relevant.

### WB analysis of CK2 protein expression in NHL cell lines

**Figure 3:**
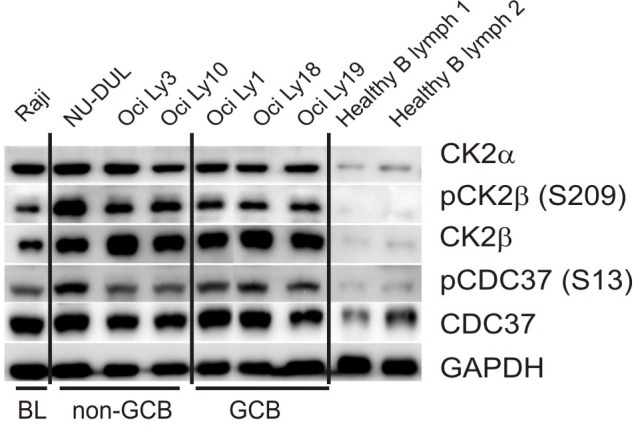
WB assays for CK2 expression in lymphoma cell-lines and normal B-cells Expression of CK2α, CK2β, CK2β phosphorylation on Ser209, total CDC37, CDC37 phosphorylation on Ser13 in BL (Raji), non-GCB type DLBCL (NU-DUL, Oci Ly 3 and Oci Ly 10) and GCB type DLBCL (Oci Ly 1, Oci Ly 18 and Oci Ly 19) cell-lines. Normal B lymphocytes (1 and 2) isolated from buffy coat of healthy donors were used as controls.

To further confirm these findings, we performed WB analysis of CK2α and CK2β expression in BL and DLBCL cell-lines. Peripheral CD19-positive B-cells from healthy donors were used as controls (Figure 3). WB analysis confirmed CK2α and CK2β over-expression in all the examined DLBCL and BL lines, with much higher protein levels than in normal B-lymphocytes. Importantly, WB also documented CK2β Ser209-phosphorylation, which is a mark of CK2 activation by Cdc2/CyclinB kinase [23], in all the cell-lines. Moreover, the presence of a phosphorylated CK2-target such as Ser13 on the HSP90-cochaperone CDC37 [24, 25] in all the examined cell-lines provided clear evidence that CK2 is also functionally active in DLBCL and BL cells (Figure 3).

### Effects of CK2 inhibition with CX4945 on NHL cell line survival and viability

**Figure 4:**
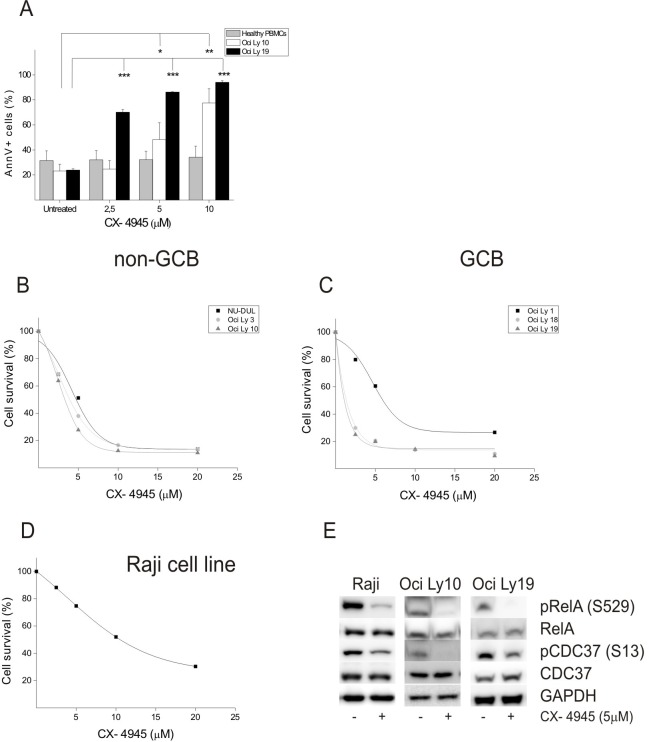
Cell viability assays on BL and DLBCL cell-lines (A) Graph summarizing the percentage of Annexin V-positive cells resulting upon exposure of healthy peripheral blood mononuclear cells (PBMC, gray bars), non-GCB type DLBCL cell line Oci Ly10 (white bars) and GCB type DLBCL cell line Oci Ly 19 (black bars) to mock (untreated) or to increasing concentrations of the CK2-selective inhibitor CX-4945. (B) MTT assay on non-GCB type DLBCL cell-lines NU-DUL, Oci Ly 3, Oci Ly 10 exposed for 48 hours to mock (100% of survival) or increasing concentrations of CX-4945. (C) and (D) Same as in B for GCB type DLBCL cell-lines Oci Ly 1, Oci Ly 18, Oci Ly 19 (C) and BL cell-line Raji (D). (E) WB analysis of the expression of total and phosphorylated CK2 specific targets RelA and CDC37 in BL (Raji), non-GCB type (Oci Ly10) and GCB type (Oci Ly 19) DLBCL cell lines after treatment for 3 hours with CX-4945 at 5 μM concentration.

We finally tested if CK2 could be a suitable target for harming B-cell lymphoma growth. Non-GCB type DLBCL cell-line Oci Ly10, GCB type DLBCL cell-line Oci Ly19 as well as normal peripheral blood mononuclear cells (PBMC, controls) were cultured in the presence of increasing concentrations of CX-4945 (2.5, 5 and 10 μM) and the amount of apoptosis was then measured after 24 hours by Annexin V staining and fluorescence activated cell sorting (FACS) analysis. Remarkably, while DLBCL cell-lines of both subtypes displayed a dose-dependent induction of apoptosis, PBMC from healthy donors were substantially spared by CX-4945 at the used concentrations (Figure 4A). Furthermore, cell proliferation/viability, as evaluated by the MTT (3-(4,5-dimethylthiazol-2-yl)-2,5-diphenyl tetrazolium bromide) assay, demonstrated that non-GCB (Figure 4B), GCB (Figure 4C) DLBCL cell-lines as well as BL Raji cells (Figure 4D) were sensitive - even though to a different extent - to the growth-arresting effect induced by CX-4945, in a dose-dependent fashion. Of note, WB analysis revealed a marked down modulation of the phosphorylation status of two CK2-specific target sites, namely Ser529 on the NF-κB subunit p65/RelA [26] and Ser13 on the co-chaperone CDC37, in Raji, Oci Ly 10 and Oci Ly 19 cell lines, after treatment for three hours with 5 μM CX-4945 (Figure 4E).

## DISCUSSION

NHL are a composite group of B-cell lymphoproliferative disorders, encompassing both indolent and aggressive tumors [27, 28]. Despite the pathobiological and clinical features of such lymphomas have been thoroughly investigated, prognosis and therapeutic outcomes remain highly variable and the search for novel therapeutic targets is still eagerly ongoing [29-31]. The recent development of protein kinase CK2 inhibitors as anti-cancer agents [15, 32, 33] and the evidence of CK2-expression in lymphocytes of reactive germinal centers (this paper and [18]) prompted us to investigate CK2 in a large series of FLs, DLBCLs and BLs.

Our results clearly demonstrate that CK2 is widely expressed in the vast majority of the commonest subtypes of NHL. Of note, immunohistochemical and cDNA arrays analyses indicate a trend for BL as associated with higher CK2 protein levels compared to both FL and DLBCL (Figure 1 and Figure 2). These data achieved on patients specimens were further sustained by *in vitro* assays on BL and DLBCL cell-lines. In particular, WB analysis revealed constitutive CK2α and CK2β expression in all the lymphoma cell-lines used, with more abundant protein levels than in normal B-lymphocytes (Figure 3). WB also provided hints about the functional status of CK2. The detection of CK2β phosphorylation on Ser209 may indeed be regarded as a compelling readout of CK2 activation by the Cdc2/CyclinB complex, a feature likely to be linked to high cell proliferative activity [23]. In addition, we detected the presence of a known specific phosphorylated target of CK2 (phospho-Ser13 on CDC37) in lymphoma cells, which provides further evidence of CK2 active status as compared to normal B cells. Interestingly, Ser13-phosphorylated CDC37 is a critical co-chaperone of HSP90, which has been clearly shown to be essential for proper folding of HSP90 client oncogenic kinases [34]. To note, CK2 mRNA and protein levels do not follow a perfectly identical trend, since we observed less marked differences in the expression of CK2 protein compared to mRNA across the different NHL subtypes. This could be due to various possibilities, for instance to the fact that the transcription of CK2 mRNAs is differently regulated in BL as compared to FL and DLBCL because of different sets of transcription factors. Also, regardless the mRNA levels, the CK2 subunits could be post-translationally controlled in neoplastic B cells, causing their differential levels within NHL subtypes. Further work will elucidate this aspect.

From a practical viewpoint, the widespread expression of CK2 limits its applicability to the routine histopathological diagnosis of B-cell lymphomas. On the other hand, the high levels and the constitutive activation of CK2 pinpoint such kinase as an additional potential target for the treatment of FL, DLBCL and BL. This hypothesis, at least for DLBCL and BL, is sustained by the results of cell viability assays after incubation with the CK2-inhibitor, CX-4945 (Figure 4). In such assays, lymphoma cell-lines demonstrated a dose- and time-dependent response to CX-4945. Remarkably, CX-4945 efficacy was supported by a clear down modulation of phospho Ser529 RelA and phospho Ser13 CDC37, two well-known CK2 targets. Regarding this latter molecule, since it is an essential co-chaperone of HSP90, it will be interesting to test whether double targeting of HSP90 and CK2 would be synergic in causing NHL cell death, similarly as in MM cells, like we recently demonstrated in *in vivo* and *in vitro* models [20].

In conclusion, our work suggests that further investigations on the pre-clinical and clinical applications of CK2-inhibitors against DLBCL, BL and possibly FL are worth pursuing. It also point to the rationale of performing parallel studies on the role of CK2-regulated networks in B-cell physiology and in B-cell tumor pathogenesis.

## MATERIALS AND METHODS

### Tissue samples

Cases were retrospectively collected from the archives of the Hematopathology Section, Sant' Orsola-Malpighi Hospital (University of Bologna, Bologna, Italy). Overall, 127 NHLs were considered: (i) 18 cases of sporadic BL; (ii) 52 cases of DLBCL (30 cases of GCB-type and 22 cases of non-GCB type, as sub-classified according to Hans' algorithm [35]); (iii) 57 cases of FL (28 cases of Bcl2-positive FL; 29 cases of Bcl2-negative FL). The present series was enriched in Bcl2-negative FLs to specifically investigate possible differences in CK2-expression between Bcl2-positive and Bcl2-negative cases. Distribution of FLs according to the histological grade was as follows: (i) 13 cases of Grade 1 FL (Bcl2-positive: 6 cases; Bcl2-negative: 7 cases); (ii) 26 cases of Grade 2 FL (Bcl2-positive: 12 cases; Bcl2-negative: 14 cases); (iii) 18 cases of Grade 3 FL (Bcl2-positive: 10 cases; Bcl2-negative: 8 cases; all cases were Grade 3A).

For each lymphoma subtype, tissue microarray (TMA) blocks were prepared, obtaining three tissue cores from each original sample. Adequate positive (lymphoid and thymic tissue) and negative (myocardium) controls were also included.

None of the patients received radiation therapy or chemotherapy prior to biopsy sampling and histological evaluation. The institutional and international (Declaration of Helsinki) ethics regulations governing research conducted on human tissues were followed, and all patients gave their informed consent.

### Immunohistochemical analysis

Immunohistochemistry was performed on 4 μm-thick FFPE sections, using CK2α (EP1963Y, Epitomics, CA, USA) and CK2β (6D5, Santa Cruz Biotechnology, CA, USA) monoclonal antibodies. Heat/EDTA-based antigen retrieval methods were applied, as previously described [18, 36]. Antigen detection was performed with the Bond Polymer Refine Detection kit in an automated immunostainer (Bond maX, Menarini, Italy) [18].

CK2α and CK2β immunostain was semiquantitatively scored in a four-tiered scale, as follows: score 0 = negative staining; score 1+ = weak positivity or positive staining in <5% of tumor cells; score 2+ = moderate positivity or strong positive staining in <50% of cells; score 3+ = strong positive staining in >50% of tumor cells. Immunohistochemical reactions were independently scored by two investigators (MP and PB; agreement k>0.8). In case of discrepancies, a consensus opinion was rendered. For the statistical interpretation of immunohistochemical data, scores 0/1+ and 2+/3+ were lumped in “low-expression” and “high-expression” groups, respectively.

### cDNA microarray analysis

The Oncomine database and gene microarray analysis tool, a repository for published cDNA microarray data (http://www.oncomine.org) [21] was explored (on September-November 2014) for CK2α, CK2α' and CK2β mRNA expression (searched genes: *CSNK2A1*, *CSNK2A2* and *CSNK2B*, respectively). Only studies assessing gene expression profiling of BL, FL and DLBCL were considered. Studies considering ≤5 cases for each lymphoma entity were excluded. Of the publicly available data sets, one met such inclusion criteria [22].

### Chemicals

CX-4945 was purchased from Activate-Scientific GmbH, Germany. The specificity and mechanism of action of this inhibitor were previously characterized [18, 37].

### Cell cultures and healthy controls' cells

Non-GCB type DLBCL cell-line NU-DUL and GCB type DLBCL cell-lines OCI Ly1 and OCI Ly18 were purchased from the Deutsche Sammlung von Mikroorganismen und Zellkulturen (DSMZ, Germany). Non-GCB type DLBCL cell-lines OCI Ly3, OCI Ly10 and GCB type DLBCL cell-line OCI Ly19 were a kind gift of Dr. F. Bertoni, Bellinzona, Switzerland. NU-DUL were maintained in RPMI 1640 medium supplemented with 15% fetal bovine serum (FCS) + 2-Mercaptoethanol (50mM; Invitrogen); OCI Ly10, OCI Ly3 and OCI Ly1 in IMDM 20% FCS + 2-Mercaptoethanol (50mM); OCI Ly18 in RPMI 10% FCS + 2-Mercaptoethanol (50mM) and OCI Ly19 in RPMI 10% + 2-Mercaptoethanol (50mM) + MEM NEAA + sodium pyruvate. BL Raji cell-line was purchased from ATCC and cultured in RPMI 20% FCS. Testing for Mycoplasma infection was carried at a monthly basis. Peripheral blood mononuclear cells (PBMCs) and B lymphocytes from healthy donors were obtained from peripheral blood as per standard Ficoll Paque^®^ protocol; B lymphocytes were purified using CD19-coated magnetic MicroBeads according to the manufacturer's protocol (Miltenyi Biotech).

### WB and antibodies

Whole cell extracts (WCE) were obtained by lysis with 20 mM Tris (pH 7.5), 150 mM NaCl, 2 mM EDTA, 2 mM EGTA supplemented with 0,5% Triton X-100 (Sigma-Aldrich), protease inhibitor cocktail (Sigma-Aldrich), phosphatase inhibitor cocktail (Thermo Scientific), 1 mM phenyl-methyl-sulfonyl fluoride (PMSF; Sigma-Aldrich), 1 μM okadaic acid (Sigma-Aldrich), dithiothreitol (DTT; Fluka). Twenty to 50 μg of WCE were subjected to SDS-PAGE, transferred to PVDF membranes and immunoblotted with the following primary antibodies: anti-CK2α (kindly provided by Dr S. Sarno, University of Padua, Italy); anti-CDC37 (Santa Cruz Biotechnology, Santa Cruz, CA) anti-phospho-Ser13 CDC37 (Abcam); anti-CK2β (BD Biosciences, USA); anti-phospho-Ser209 CK2β (Assay Biotech) and anti-GAPDH (Ambion); anti-RelA (Abcam) and anti-phospho-Ser529 RelA (Santa Cruz Biotechnology, Santa Cruz, CA). As secondary antibodies: anti-rabbit IgG HRP-linked antibody (Cell Signaling, Beverly, MA); HRP labeled goat anti-mouse IgG (KPL, Gaithersburg, MD, USA). Detection was performed using ECL (Pierce, Thermo Scientific), LiteAblot Extend Long Lasting Chemiluminescent Substrate (Euroclone) or LiteAblot Turbo Extra Sensitive Chemiluminescent Substrate (Euroclone) according to manufacturer's instructions.

### Determination of apoptosis

Apoptosis was assessed by Annexin V/Propidium Iodide staining according to the manufacturers' instructions (BD Pharmingen). After 24 hours of treatment cells were labeled with FITC-conjugated Annexin V and Propidium Iodide (Becton-Dickinson, Italy). Fluorescence Activated Cell Sorting (FACS) analysis was performed using a FACS-Canto Cell Cytometer and the CellQuest software (Becton-Dickinson, Italy).

### MTT assay

MTT (3-(4,5-dimethylthiazol-2-yl)-2,5-diphenyl tetrazolium bromide) assay was performed in 96-well plates using a CellTiter 96^®^ kit (Promega). Briefly, 0.5 × 10^4^ cells were plated in 96-well plates the day before the experiment. At the end of the treatment with CX-4945 (48 hours), culture medium was removed and replaced with 100 ml of complete medium without phenol red + 50 ml of MTT to each well. During an incubation period of 1.5 hour at 37°C, the MTT salt is metabolically reduced only by viable cells into an insoluble coloured formazan; the absorbance/optical density was read and reported by a microplate reader at 570 nm. Values are expressed as percentage of viable cells compared with untreated cells, considered as 100%. This assay was performed in parallel with all cell experiments to exclude any artefact resulting from differences in cell viability.

### Statistical analysis

Data were evaluated for their statistical significance with the two-tail paired Student's t test and differences and correlations between groups were tested by applying analysis of variance (ANOVA) or the Fisher's exact test (immunohistochemical results). Pearson's product moment correlation test was also adopted in a specific analysis. *P* values below 0.05 were considered statistically significant.

## SUPPLEMENTARY MATERIAL, FIGURES


