# Supplementary material for: Protein kinase CK2 is widely expressed in follicular, Burkitt and diffuse large B-cell lymphomas and propels malignant B-cell growth
Source: Oncotarget. 2015 Jan 31;6(9):6544–52. doi: 10.18632/oncotarget.3446 (PMC4466633; doi:10.18632/oncotarget.3446)
Supplement: Supplementary file 1 [file oncotarget-06-6544-s001.pdf]

Protein kinase CK2 is widely expressed in follicular, Burkitt and Diffuse Large B-cell Lymphomas and propels malignant B-cell growth

SUPPLEMENTARY MATERIAL

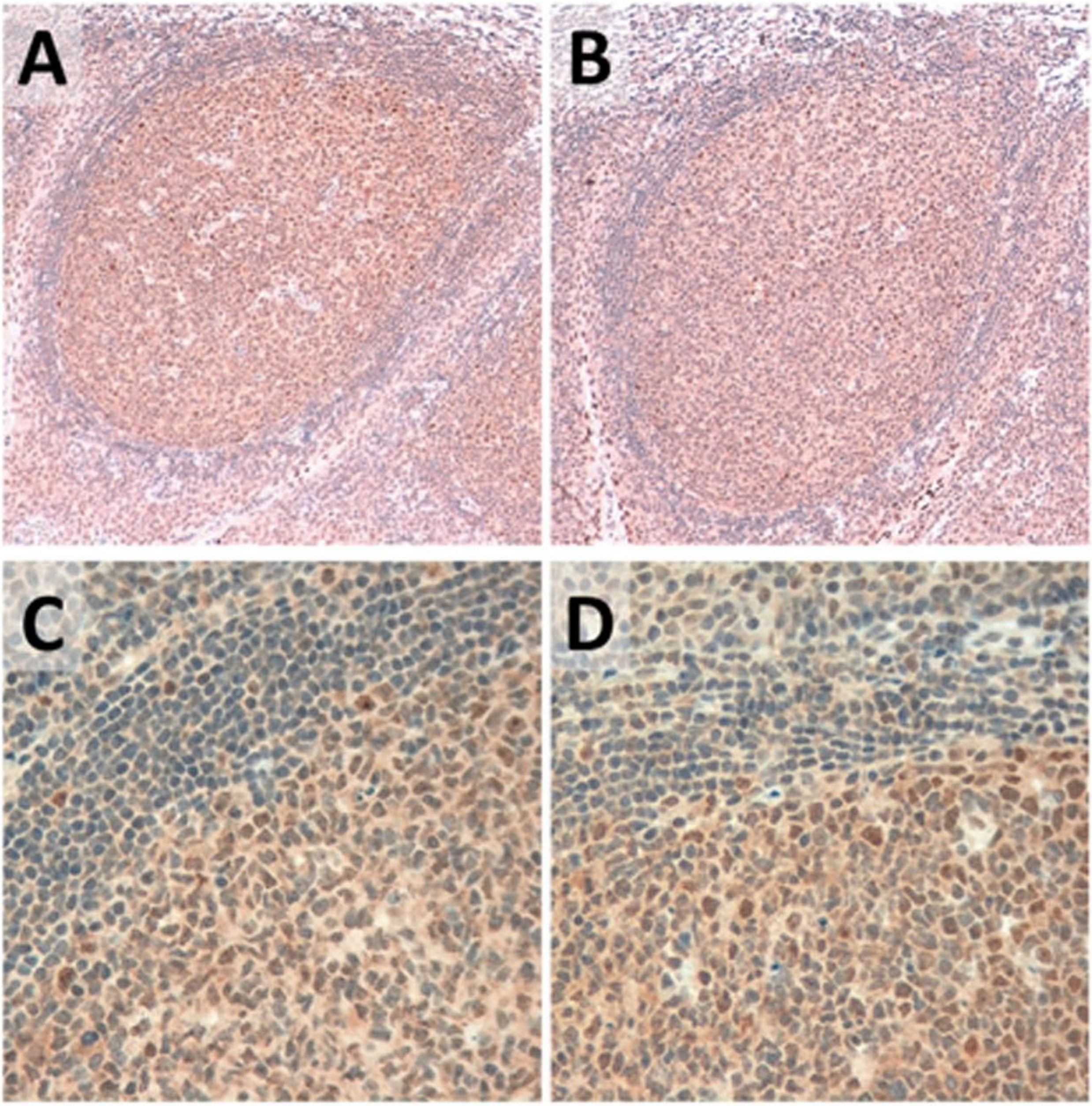

Supplemental Figure 1: CK2α and CK2β Immunohistochemical expression in non-neoplastic lymphoid tissue. CK2α (**A,C**) and CK2β (**B,D**) expression in normal tonsil showing positivity of centrocytes and centroblasts of reactive germinal centers. Mantle zone B-cells are consistently negative for both markers (Immunoperoxidase stain; original magnification, x5 and x20).

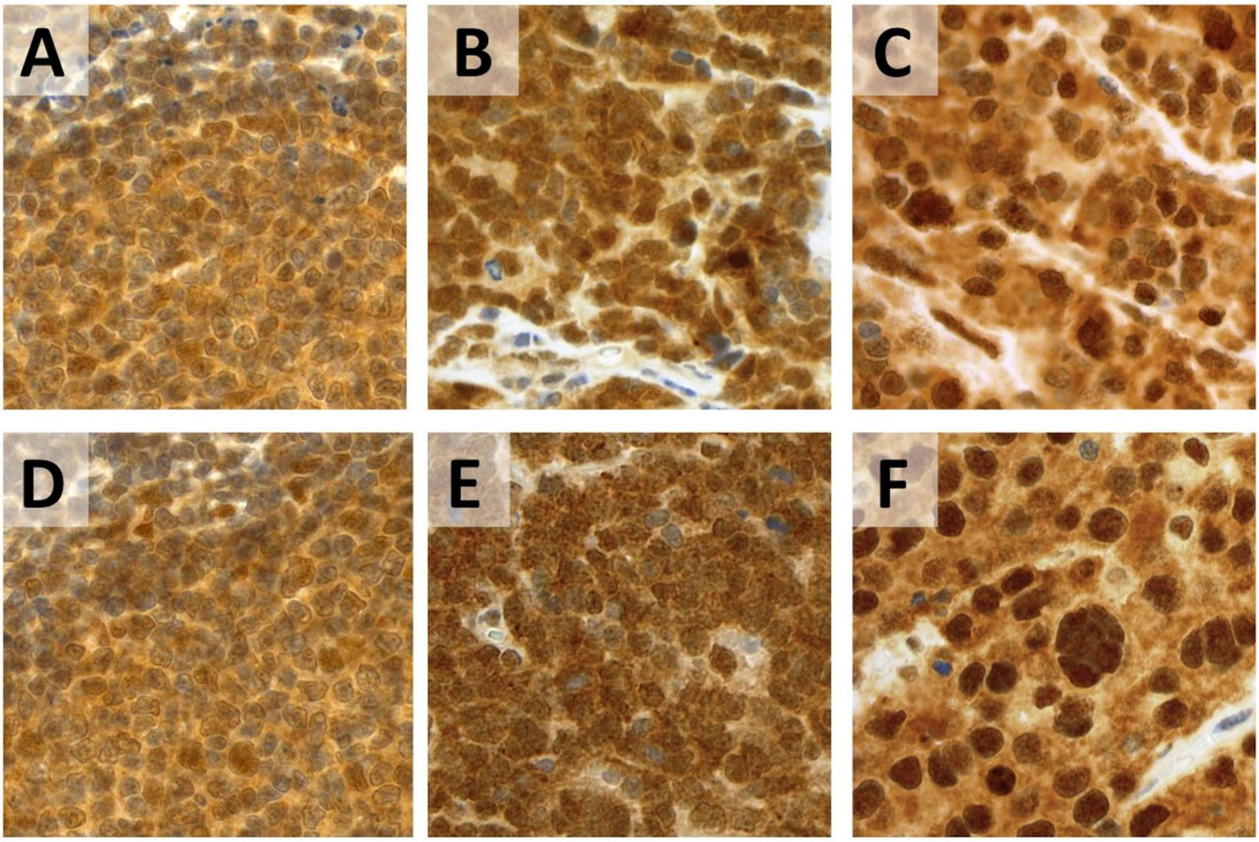

Supplemental Figure 2: Nuclear and Cytoplasmic expression of CK2α and CK2β in Follicular, Burkitt and Diffuse Large B-Cell Lymphoma. (A-C) Representative examples of CK2α score 3+ immunostain in Follicular Lymphoma (**A**), Burkitt Lymphoma (**B**) and Diffuse Large B-cell Lymphoma (**C**);. in all cases, cytoplasmic and nuclear expression is observed. **(D-F)** CK2β immunostain in Follicular Lymphoma (**D**), Burkitt Lymphoma (**E**) and Diffuse Large B-cell Lymphoma (**F**) showed similar results with nuclear and cytoplasmic protein expression (Immunoperoxidase stain; original magnification, x40).
